# T175. A 10-YEAR LONGITUDINAL STUDY OF GREY MATTER VOLUME IN FIRST EPISODE OF NON-AFFECTIVE PSYCHOSIS

**DOI:** 10.1093/schbul/sby016.451

**Published:** 2018-04-01

**Authors:** Diana Tordesillas-Gutiérrez, Ayesa-Arriola Rosa, Victor Ortiz-García de la Foz, Esther Setien-Suero, Javier Vázquez-Bourgon, Benedicto Crespo-Facorro

**Affiliations:** 1 Valdecilla Biomedical Research Institute IDIVAL; 2 Marqués de Valdecilla University Hospital, IDIVAL, CIBERSAM; 3 University Hospital Marques de Valdecilla – IDIVAL, University of Cantabria, CIBERSAM

## Abstract

**Background:**

Structural abnormalities in First Episode of Non- Affective Psychosis (FEP) are shown to be present at the time of onset of the illness. Although there are multiple cross-sectional studies in chronic patients there is no clear evidence how these alterations progress years after the appearance of the first episode.

**Methods:**

Data for the present investigation were obtained from an ongoing epidemiological and longitudinal intervention programme of first-episode psychosis (PAFIP) conducted at the Marqués de Valdecilla University Hospital (HUMV), Spain. Images for 62 FEP patients and 47 healthy controls were acquired at baseline and 10 year follow-up on the same 1.5-T whole-body scanner (SIGNA, GE, Milwaukee, WS, USA). Three-dimensional T1-weighted images, using a spoiled gradient-recalled acquisition in the steady state (GRASS) (SPGR) sequence, were acquired in the coronal plane with the following parameters: TE=5 msec, TR =24 msec, NEX=2, rotation angle =45°, FOV= 26 x 19.5 cm, slice thickness =1.5 mm and a matrix of 256 x 192.

Structural imaging data for each subject was analyzed using serial longitudinal Statistical Parametric Mapping software (SPM12). After segmenting the mid-point average and multiply the result by the jacobian maps, DARTEL was applied to spatially normalise de diferences. T-test between both groups was performed, allowing voxel-wise comparison of progressive structural change. All results were p<0.05 FWE corrected.

**Results:**

FEP patients exhibited progressive bilateral atrophy of the anterior cingulate bilaterally, the right inferior orbital, middle and superior frontal giri, left precentral and postcentral giri and cerebellum. We found no areas were grey matter was greater in controls than in patients.

**Discussion:**

In this study we analyze a well characterized sample of patients with a first episode of non-affective psychosis in the first weeks after onset and 10 years later. Our results confirm that, apart from the grey matter volume reduction presented at baseline, patients show a progressive grey matter loss in anterior cingulate, frontal and parietal lobes as well as cerebellum.

